# Elucidation of Sulforaphane‐Mediated Effects on the Cellular Human Metabolome Using Metabolic Profiling

**DOI:** 10.1002/mnfr.70373

**Published:** 2026-01-16

**Authors:** Nadine Bieß, Hans‐Ulrich Humpf, Matthias Behrens, Andrea Gerdemann

**Affiliations:** ^1^ Institute of Food Chemistry University of Münster Münster Germany

**Keywords:** HepG2, HPLC‐MS/MS, metabolic profiling, *N*‑ethylmaleimide, sulforaphane

## Abstract

Sulforaphane (SFN) is an isothiocyanate derived from glucoraphanin, which occurs in broccoli. Several human health‐promoting effects are attributed to the consumption of cruciferous vegetables or food supplements containing SFN. Its described cancer‐preventive, chemoprotective, and antioxidant properties made SFN an increasingly important research topic. The antioxidant properties have previously been connected to stimulation of the Nuclear factor erythroid‐2‐related factor 2 (Nrf2)/Kelch‐like ECH‐associated protein 1 (Keap1) signaling pathway. However, the global effects of SFN on the primary metabolic pathways have yet to be fully unraveled. Therefore metabolic profiling was used to elucidate the effects of SFN on the cellular metabolome. For this purpose, human hepatoblastoma cells (HepG2) were incubated with SFN and the changes of primary metabolite levels were determined by targeted hydrophilic interaction liquid chromatography tandem mass spectrometry (HILIC‐MS/MS) analysis. Metabolic profiling revealed that SFN affects the tricarboxylic acid cycle, the urea cycle and their related amino acids. Furthermore, effects on glycolysis, pentose phosphate pathway and glutathione (GSH) levels were observed. This profound impact on nearly all primary metabolic pathways indicates a high bioactive potential of this natural compound. Especially elevated GSH levels underline the commonly described antioxidant potential of SFN.

## Graphical abstract

Sulforaphane (SFN) is an isothiocyanate derived from broccoli and is related to various health‐promoting effects. By using metabolic profiling on the hepatoblastoma cell line HepG2 several effects on pathways such as TCA cycle, urea cycle and redox metabolism through SFN were identified.

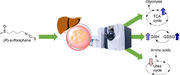

**Abbreviations**
6‐PG: 6‐phosphogluconate
ATP: adenosine triphosphate
CDP: cytidine diphosphate
CoA: coenzyme A
CoA‐NEM: coenzyme A *N*‑ethylmaleimide derivative
CPS I: carbamoyl‐phosphate synthetase 1
CTP: cytidine triphosphate
FCS: fetal calf serum
G6PD: glucose 6‐phosphate dehydrogenase
GCL: glutamate cysteine ligase
Glycerol‐3‐P: glycerol 3‐phosphate
GSH: glutathione reduced
GS‐NEM: glutathione *N*‑ethylmaleimide derivative
GSSG: glutathione disulfide
Hexose‐P: hexose phosphates
HILIC: hydrophilic interaction liquid chromatography
ITCs: isothiocyanates
Keap1: Kelch‐like ECH‐associated protein 1
NADPH: nicotinamide adenine dinucleotide phosphate
NEM: *N*‑ethylmaleimide
Nrf2: nuclear factor erythroid‐2‐related factor 2
Pentose‐P: pentose phosphates
PPP: pentose phosphate pathway
ROS: reactive oxygen species
SFN: sulforaphane
TCA cycle: tricarboxylic acid cycle
UDP‐Glucose: uridine diphosphate glucose

## 1 Introduction

**FIGURE 1:**
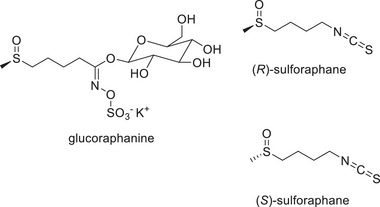
Chemical structures of glucoraphanin and the isomers of sulforaphane.

The consumption of cruciferous vegetables is associated with a healthy human diet. In addition to other beneficial compounds, cruciferous vegetables contain glucosinolates, which can be enzymatically converted by myrosinases to isothiocyanates (ITCs). Since these enzymes are present in different compartments of plant cells, this reaction only occurs after tissue disruption [1]. It is evident that the human gut microbiota can also convert glucosinolates into ITCs, with the conversion rate depending on the composition of the microbiota [2]. A highly researched ITC is sulforaphane (SFN). The glucosinolate glucoraphanin, found in broccoli, is exclusively converted into the (*R*)‐enantiomer of SFN, making (*R*)‐SFN the naturally occurring isomer (Figure 1) [3]. In broccoli sprouts, even higher concentrations of glucoraphanin can be found than in mature broccoli [4].

After consumption, SFN is well absorbed from the jejunum [5] and then metabolized through the mercapturic acid pathway in the liver [6]. Metabolites originating from this pathway are detectable in plasma and are excreted through urine and feces [2, 7].

Due to its various health‐related effects, research on SFN has become increasingly important. SFN is widely discussed for its cancer‐preventive and chemoprotective properties, which have been observed in epidemiological studies [8]. In rats, SFN showed a dose‐dependent reduction in tumor number and size, and even delayed their appearance. The induction of apoptosis, cell cycle arrest, and DNA damage in cancer cells have been discussed as mechanism underlying the cancer‐preventive properties of SFN [9, 10]. Another explanation is the antioxidant property of SFN, which has been broadly discussed in the literature. Reactive oxygen species (ROS) increase the risk of mutations and neoplasia. Therefore, detoxification of ROS by antioxidant metabolites may result in reduced cancer risk [11]. SFN is known to stimulate the Nuclear factor erythroid‐2‐related factor 2 (Nrf2)/Kelch‐like ECH‐associated protein 1 (Keap1) signaling pathway, which causes the biosynthesis of cytoprotective enzymes such as glutathione *S*‐transferases, UDP‐glucuronosyl transferases, NAD(P)H:quinone oxidoreductase 1, and glutamate cysteine ligase (GCL) [12, 13]. Glutathione (GSH), whose synthesis is upregulated through Nrf2/Keap1 signaling, plays a vital role in the antioxidant and cytoprotective mechanism of cells. Due to its reactivity, reliable analysis of GSH levels is challenging. In order to prevent oxidation/dimerization to glutathione disulfide (GSSG) during sample processing, a derivatization method using *N*‐ethylmaleimide (NEM) was developed previously [14, 15].

The impact of SFN on the metabolism of the hepatoblastoma cell line HepG2 was analyzed in a previous study and was related to different glucose contents in the cell culture medium. The researchers used different methods such as RNA sequencing, GC‐MS with stable isotope tracers, and HPLC‐MS [16]. This study demonstrated the antioxidant properties of SFN through Nrf2/Keap1 stimulation, leading to elevated GSH and nicotinamide adenine dinucleotide phosphate (NADPH) levels, which was accompanied by a decrease of glutamine, cysteine, and glycine levels. However, GSH was determined without derivatization, possibly leading to higher variances and potential GSH oxidation during sample preparation. Following the identification of elevated GSH levels as the primary metabolic alteration, the study focused predominantly on metabolic alterations associated with GSH synthesis. As an indication of the redirection of glutamine toward GSH synthesis the altered levels of glutamine, glutamate, succinate, and fumarate were examined. Additionally, metabolites such as serine and glycine—linked to 1C metabolism—and glucose 6‐phosphate and ribose 5‐phosphate—associated with glycolysis/pentose phosphate pathway (PPP)—were discussed in relation to GSH synthesis. Consequently, additional potential effects of SFN on the metabolome may have been overlooked, and global impact of SFN on cellular metabolism remains incompletely characterized. However, the study did not specify which metabolites were analyzed besides those explicitly discussed [16].

To achieve a more complete characterization of the effects of SFN on cellular metabolism and to achieve more reliable results concerning GSH levels, a metabolic profiling method based on targeted hydrophilic interaction liquid chromatography (HILIC)‐MS/MS and complemented by NEM derivatization was employed. Metabolic profiling can be used to determine effects of xenobiotics on the cellular metabolome. The xenobiotics’ effects on cellular metabolites such as carbohydrates, amino acids, small organic acids, and nucleic acid derivatives help to interpret xenobiotic bioactivity and discover toxicological endpoints with higher precision [17, 18]. Robust interpretation of metabolic alterations requires accurate identification and quantification of metabolites. The targeted approach is advantageous in this context, as it offers lower limits of detection and quantification and higher selectivity compared to an untargeted method. The employed metabolic profiling method comprises more than 100 metabolites, which cover the main metabolic pathways [18]. Therefore, metabolic profiling is a powerful tool to identify the effect of SFN with a single method and provides an overview of the primary metabolism. Given the liver's central role in xenobiotics metabolism and the fact that SFN is metabolized through the mercapturic acid pathway in the liver [6], HepG2 cells are employed as in vitro model organism for human hepatocytes. In this study, we complemented our metabolic profiling method by thiol derivatization with NEM according to a previous study [15] and use this improved method to determine metabolic alterations caused by SFN on HepG2 cells.

## 2 Experimental Section

### 2.1 Chemicals and Reagents

The HPLC‐MS grade solvents were obtained from Fisher Scientific (Schwerte, Germany). For purified water generation a PureLab Flex2 system (Veolia Water Technologies, Celle, Germany) was used. Ammonium acetate and ammonia (25%, v/v), which were used as solvent additives, were obtained from VWR (Darmstadt, Germany) and Grüssing (Filsum, Germany), respectively.

The internal standards for metabolic profiling, d‐(‐)‐*α*‐phenylglycine and vanillic acid, were purchased from Sigma‐Aldrich (Steinheim, Germany), caffeine was purchased from Merck (Darmstadt, Germany). *N*‐ethylmaleimide (NEM), used for derivatization, was obtained from Sigma‐Aldrich (Steinheim, Germany). ( *r* / *s* )‐sulforaphane (purity > 94%, SFN) and ( *r* )‐sulforaphane (purity > 95%) were both purchased from Biosynth (Bratislava, Slowakia). Due to the cheaper price the racemic mixture (*R*/*S*)‐SFN was used for some basic experiments.

### 2.2 Cell Culture

The hepatoblastoma cell line HepG2 was obtained from American Type Culture Collection (ATCC, Manassas, USA). The cells were cultured using Dulbecco's Modified Eagle Medium (DMEM, high glucose, with glutamine, Gibco, Prat de Lloregat, Spain), which was supplemented with 10% (v/v) fetal calf serum (FCS, PAN Biotech, Aidenbach, Germany), 10 mM *N*‐2‐hydroxyethylpiperazine‐*N′*‐2‐ethanesulfonic acid (HEPES buffer, Carl Roth, Karlsruhe, Germany), and 100 U/mL penicillin and 100 µg/mL streptomycin (PAN Biotech, Aidenbach, Germany). The cells were cultured at 37°C and in a humidified CO_2_‐atmosphere (5%). Subculturing was done every 7 days and the medium was exchanged twice a week.

## 3 Chemical Stability of Sulforaphane in Cell Culture Medium

### 3.1 Experimental Design for Chemical Stability Analysis

To determine chemical stability, ( *r* / *s* )‐SFN was incubated for 24 h under cell culture conditions (37°C, 5% CO_2_) without cells. For this experiment, ( *r* / *s* )‐SFN was diluted in FCS‐free DMEM or thiol‐free DMEM (high glucose, without glutamine, methionine, and cysteine, Gibco, Prat de Lloregat, Spain) supplemented with 2 mM glutamine (USP for biochemistry, Carl Roth, Karlsruhe, Germany). For some experiments, the medium was additionally supplemented with 0.2 mM methionine (Merck, Darmstadt, Germany). As a control for the volatility of SFN, an experiment with phosphate‐buffered saline (PBS, PAN Biotech, Aidenbach, Germany) was also carried out. The stability of SFN was tested at 100 nM, 1 µM, and 10 µM (*n* = 2). These concentrations were chosen to cover a large concentration range and to determine, whether there is a relation between concentration and degradation rate. Before incubation, an aliquot of the solutions to be tested was frozen until analysis as a non‐incubation control sample.

### 3.2 Untargeted HPLC‐HRMS Analysis of Sulforaphane

After incubation, all samples were analyzed by untargeted HPLC coupled to high resolution MS. For chromatographic separation, a Nucleodur C18 Gravity SB column (2 mm × 100 mm, 1.8 µm, Macherey‐Nagel, Düren, Germany) was used with an ACN/water gradient and 0.1% formic acid as additive in both solvents. The HPLC instrumentation consisted of an Elute HT Pump HPG 1300 (Bruker Daltonics, Bremen, Germany), an Elute Column Oven (Bruker Daltonics, Bremen, Germany) and a PAL HTC‐xt autosampler (CTC analytics, Zwingen, Switzerland). The mass spectrometric analysis was carried out on a quadrupole TOF‐MS with an Apollo II ESI source (Impact II, Bruker Daltonics, Bremen, Germany). Detailed measurement parameters can be found in Table S1.

### 3.3 Data Processing for Chemical Stability

The chemical stability raw data was processed using Compass Data Analysis 4.4 (Bruker Daltonics, Bremen, Germany) and Skyline 22.2 (University of Washington, Seattle, USA). The integrated peak areas were further processed using OriginPro 2024 (OriginLab Corporation, Northhampton, MA, USA). The peak area of SFN in the incubated sample was normalized to the peak area of SFN in the respective frozen control sample. The mean and the deviation of individual values from the mean were calculated (*n* = 2).

## 4 Metabolic Profiling of Sulforaphane

### 4.1 Treatment of HepG2 Cells With Sulforaphane

For metabolic profiling, 3 × 10^6^ HepG2 cells were seeded in cell culture dishes with 10 cm diameter. Three different cell passages of the HepG2 cells were used, and the cells were seeded in three cell culture dishes for each cell passage, resulting in three biological replicates, with three technical replicates each (*n* = 3 × 3). 24 h after seeding, the DMEM containing FCS was replaced by FCS‐free DMEM. After an additional 24 h, the cells were either incubated with sub‐cytotoxic 10 µM ( *r* )‐SFN or 1% DMSO, which served as a solvent control, both in thiol‐containing, FCS‐free DMEM. The cells were treated for another 24 h. The enantiomerically pure ( *r* )‐sfn was used, because it is the naturally occurring enantiomer [3]. The concentration was selected based on a resazurin reduction assay, which was carried out according to a previous publication [19]. The results can be found in Figure S1. To compare also metabolic effects of thiol deprivation, an additional experiment was carried out, treating the cells with thiol‐free DMEM containing methionine (0.2 mM). This experiment was performed without the addition of SFN.

### 4.2 Sample Processing With *N*‐Ethylmaleimide Derivatization

Further sample processing was done according to a previous publication by Gerdemann et al. [18] with an additional NEM derivatization for reproducible GSH determination. At the end of the incubation time, the cells were washed with PBS and layered with 500 µL refrigerated (8°C) extraction solvent, consisting of ACN/water (4+1, v/v) with 10 mM NEM for derivatization as well as 50 µM d‐(‐)‐*α*‐phenylglycine, 100 µM caffeine, and 100 µM vanillic acid as internal standards. Samples were further processed immediately, and cell culture dishes were maintained at 8°C throughout the processing procedure. Cells were detached using a sterile cell scraper (Corning Science, Berlin, Germany). The cell culture dish was washed once with 500 µL of the refrigerated extraction solvent containing the internal standards and NEM, and was then washed with 500 µL refrigerated ACN/water (4+1, v/v) with 10 mM NEM but without internal standards. The suspensions were combined in 2 mL Safe‐Lock tubes (Eppendorf, Hamburg, Germany). The following sample processing steps such as cell lysis and extraction were carried out according to Gerdemann et al. [18]. After vacuum drying, the residue was reconstituted in 200 µL ACN/water (1+1, v/v) and frozen overnight. If there was insoluble residue, the samples were centrifuged (10 min, 20°C, 860 × *g*) and the supernatant was then used for targeted HILIC‐MS/MS measurement.

### 4.3 Targeted HILIC‐MS/MS Analysis for Metabolic Profiling

The chromatographic separation was carried out using a PEEK‐lined InfinityLab Poroshell 120 HILIC‐Z column (2.1 mm × 100 mm, 2.7 µm, Agilent Technologies, Waldbronn, Germany), employing a 15 min gradient elution with solvent A consisting of ACN/water (9+1, v/v) and solvent B consisting of ACN/water (1+1, v/v). Both solvents were supplemented with 10 mM ammonium acetate and adjusted to pH 9 by adding ammonia (25%). For tandem mass spectrometry, a triple quadrupole MS (EVOQ Elite, Bruker Daltonics, Bremen, Germany) with a heated ESI source was used, equipped with an Elute HT Pump HPG 1300 (Bruker Daltonics, Bremen, Germany), an Elute Column Oven (Bruker Daltonics, Bremen, Germany), and a PAL HTC‐xt autosampler (CTC analytics, Zwingen, Switzerland), which was set to room temperature. Detailed parameters and analyte transitions are available in Tables S2 and S4.

### 4.4 Quality Control and Data Processing for Metabolic Profiling

For processing of metabolic profiling data, MS Workstation (Version 8.2.1, Bruker Daltonics, Bremen, Germany) and TASQ 2024b were used. Each metabolic profiling analysis set contained a reference cell extract and a standard mixture of several analytes (Table S3) for chromatography control purposes. The reference cell extract consisted of extracts from different cell lines [HepG2, HT‐29 (colon carcinoma cells [20]) and IHKE (immortalized human kidney epithelial cells [21])] and was used to cover non available reference compounds [18]. After peak integration, the peak areas were further processed using Microsoft Excel 2021 (Microsoft Corporation, Redmond, USA). To compensate potential loss of metabolites during sample processing, the metabolite peak areas were normalized to the internal standard (vanillic acid) peak area. Vanillic acid was incorporated alongside caffeine to improve the existing method [18] in terms of standard deviation and robustness. It was selected based on its low relative standard deviation (Table S5) and stronger retention than caffeine, therefore eluting closer to most relevant metabolites.

To calculate the fold‐change, each value was divided by the mean value of the respective solvent control and then normalized to zero. Due to the limited availability of reference compounds, these are semi‐quantitative results. For all replicates, mean values and standard deviations were calculated, and statistical analysis was done by an unpaired, heteroscedastic Student's *t*‐ test (***p ≤ 0.001, **p ≤ 0.01, *p ≤ 0.05). The heatmap and the bar graphs were drawn using OriginPro 2024 (OriginLab Corporation, Northhampton, MA, USA). The chemical structures, reactions, and pathway maps were drawn using ChemDraw Professional 23.1.2 (PerkinElmer, Shelton, CT, USA).

As the pentose phosphates (ribose 5‐phosphate, ribulose 5‐phosphate, and xylulose 5‐phosphate) cannot be fully resolved by the employed HILIC method, their peak areas were combined and referred to as pentose phosphates (pentose‐P) in the following. The same applies to the hexose phosphates fructose 6‐phosphate, glucose 1‐phosphate, and glucose 6‐phosphate, which will be referred to as hexose phosphates (hexose‐P).

## 5 Results and Discussion

### 5.1 Chemical Stability of Sulforaphane

**FIGURE 2:**
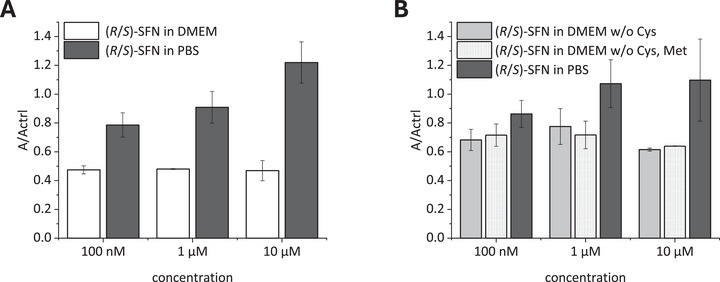
Mean relative amount of ( *r* / *s* )‐sulforaphane (( *r* / *s* )‐SFN) after 24 h of incubation in different cell culture media under cell culture conditions (37°C, 5% CO_2_) relative to the cell‐free control sample. Shown are the relative peak areas (A) of ( *r* / *s* )‐SFN analyzed by untargeted HPLC‐TOF‐MS compared to the control samples (Actrl). The error bars depict the absolute deviation from the mean value. (A) ( *r* / *s* )‐SFN (100 nM, 1 µM, 10 µM) in complete DMEM and PBS (*n* = 2, technical replicates). (B) ( *r* / *s* )‐SFN (100 nM, 1 µM, 10 µM) in DMEM without cysteine and with methionine (w/o Cys), in DMEM without cysteine and methionine (w/o Cys, Met) and in PBS (*n* = 2, technical replicates).

The instability of phytochemicals under cell culture conditions is a known challenge when used in cell‐based applications. As SFN has been described to react with free sulfhydryl groups [22], we investigated the potential impact of those medium components on its stability. Therefore, ( *r* / *s* )‐SFN was incubated in different concentrations in cysteine‐free medium, thiol‐free medium, or thiol‐containing medium for 24 h. The SFN content was determined via HPLC‐HRMS, as described in the material and methods section. The results are shown in Figure 2. Incubation with thiol‐containing DMEM resulted in a decrease of SFN concentrations up to 50% compared to the control sample, but no clear concentration dependency was apparent (Figure 2A). In comparison, the stability of SFN was improved to about 70% by using thiol‐free medium (Figure 2B). While the presence of methionine seemed to have no impact, the removal of cysteine caused the stability increase. Therefore, the methyl group of methionine hinders the reaction of SFN with the sulfur. These observed effects are consistent with previous findings, where the tendency of SFN binding to thiols has been described [22]. In the same study, the authors reported, that SFN is also able to bind to amino groups. Therefore, the isothiocyanate group can also bind to the amino acids in cell culture medium (Figure S2) [22]. However, considering that its reactivity is up to 10^4^ times lower compared to that of sulfhydryl groups, it is unlikely to account for the missing 30% [23].

### 5.2 Metabolic Alterations Caused by Cysteine Deprivation

As cysteine‐free medium resulted in increased SFN stability, we tested its suitability for metabolic profiling using targeted HILIC‐MS/MS compared to thiol‐containing medium. An overview of the metabolic alterations induced by cysteine deprivation (‐Cys) is depicted in a heatmap (Figure S3). Cysteine deprivation affects several metabolites (detailed results in Table S6). It is not surprising that cysteine deprivation results in a decrease of glutathione levels (GS‐NEM: −2.16‐fold*** and GSSG: −9.32‐fold***), as this compound is formed by cysteine with glutamate and glycine. Lee et al. reported similar results in their experiments [24]. Furthermore, cysteine deprivation causes alterations of the amino acid levels with asparagine (1.11‐fold*), histidine (0.26‐fold**), lysine (0.39‐fold**), threonine (0.37‐fold*), and serine (0.65‐fold*) being increased and cystine (−30.65‐fold***) being decreased as expected. These findings are related to previous studies, where cysteine deprivation caused differentially expressed genes involved in amino acid uptake and metabolism [24]. Further changes were observed in the levels of nucleoside derivatives, as well as increasing levels of glycerol 3‐phosphate (0.65‐fold***) and argininosuccinate (1.04‐fold***), accompanied by decrease of pentose‐P (−0.36‐fold*), *N*‐acetylglutamate (−0.76‐fold**), and succinate (−0.47‐fold***). The latter can be explained by the requirement of cysteine for the biosynthesis of coenzyme A (CoA), which serves as a precursor of succinyl‐CoA and thereby contributes to the production of succinate in the TCA cycle. However, a decrease of CoA analyzed by the derivative CoA‐NEM has not been observed. Because HepG2 cells are unable to convert methionine to cysteine, the cysteine‐deficiency cannot be compensated by adding methionine to cell culture medium [25]. Therefore and due to the significant metabolic alterations, the cysteine‐free medium is not suitable for metabolic profiling of SFN.

### 5.3 Effects of (*R*)‐Sulforaphane on the Metabolome

**FIGURE 3:**
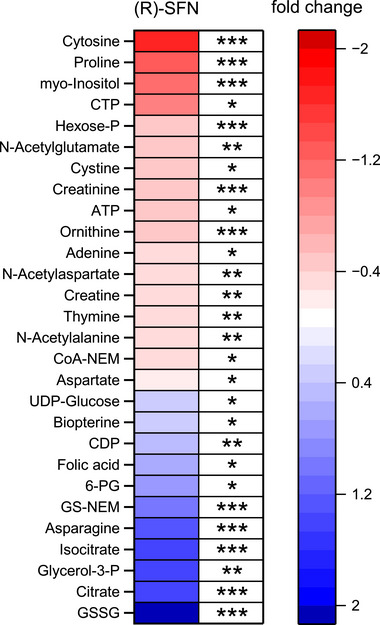
Heatmap of mean metabolic alterations analyzed by targeted HILIC‐MS/MS in HepG2 cells after 24 h (*r* )‐sulforaphane incubation ((*R*)‐SFN) illustrated as fold change to solvent control. Blue boxes indicate enrichment while red boxes indicate depletion of metabolites relative to the control sample. The significance levels are depicted as ***p ≤ 0.001, **p ≤ 0.01, *p ≤ 0.05 according to Student's *t*‐test (*n* = 3 × 3, 3 biological with 3 technical replicates each). 6‐PG, 6‐phosphogluconate; ATP, adenosine triphosphate; CDP, cytidine diphosphate; CoA‐NEM, coenzyme A N‐ethylmaleimide derivative; CTP, cytidine triphosphate; glycerol‐3‐P, glycerol 3‐phosphate; GS‐NEM, glutathione N‐ethylmaleimide derivative; GSSG, glutathione disulfide; hexose‐P, hexose phosphate; UDP‐glucose, uridine diphosphate glucose.

To determine the effects of SFN on their metabolome, HepG2 cells were incubated for 24 h with 10 µM ( *r* )‐sfn and their metabolome was analyzed by targeted HILIC‐MS/MS. Given the above‐described significant effects of cysteine deprivation on cellular metabolism, thiol‐containing DMEM was used for incubation. However, it was shown that SFN stability in this thiol‐containing medium decreased to about 50% after 24 h. Since the remaining treatment of about 5 µM was found to still possess biological activity [26] and to ensure comparability with other studies [16], the incubation time of 24 h was retained and the decrease of SFN to 50% was accepted. Consequently, the formation and biological effects of the SFN reaction products need to be addressed. Considering the composition of the cell culture medium and the reactivity of SFN toward thiol groups [22], the predominantly formed conjugate is the dithiocarbamate derivative of SFN and cysteine. A study investigating the differential effects of SFN and its metabolites, including the SFN‐cysteine conjugate, on HepG2 cells, found no significant differences in their cellular responses regarding the activation of the Nrf2/Keap1 pathway, which is described to be crucial for the cytoprotective properties of SFN [26]. SFN also exhibits reactivity toward amino groups and can therefore react with the amino acids present in the cell culture medium [22]. Additionally, SFN also exhibits some reactivity toward hydroxy groups. However, the reactivity of SFN toward amino and hydroxy groups is approximately 10^3^–10^4^ times lower than its reactivity toward thiol groups, which makes them less relevant for this experiment [23]. Consequently, the possible effects of SFN reaction products were considered minor contributors of the observed effects. The metabolic profiling results of ( *r* )‐SFN are depicted in the heatmap in Figure 3. As can be seen, ( *r* )‐SFN causes multiple alterations in different metabolic pathways (more detailed results can be found in Table S6).

### 5.4 Effects of SFN on the TCA Cycle

**FIGURE 4:**
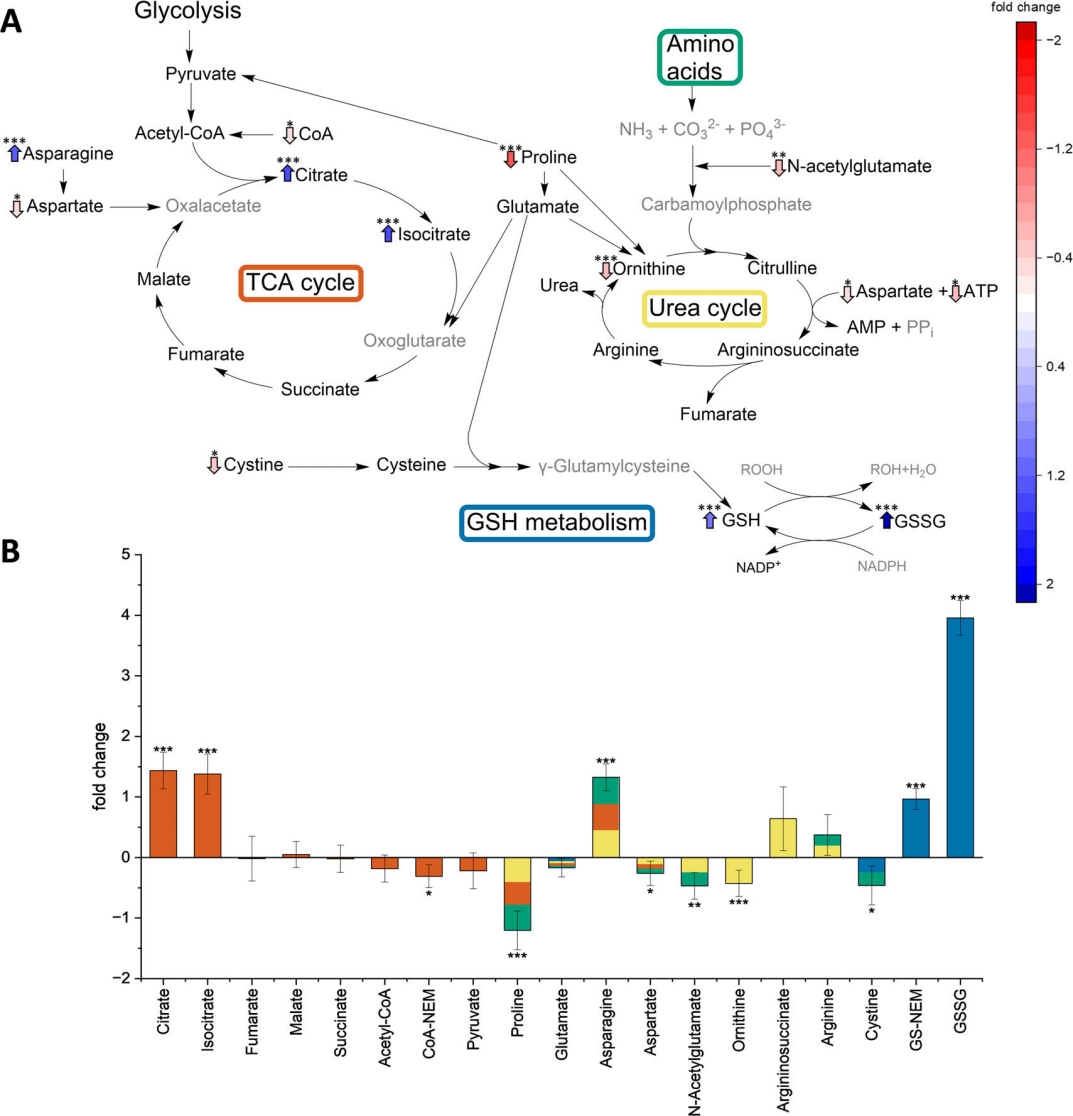
Mean metabolic alterations caused by ( *r* )‐sulforaphane (( *r* )‐SFN) in tricarboxylic acid cycle (TCA cycle), the urea cycle and the glutathione metabolism (GSH metabolism) analyzed by targeted HILIC‐MS/MS. (A) Depiction of the metabolites of the TCA cycle, urea cycle, and GSH metabolism. The colored arrows indicate the statistically significant metabolic changes with the blue up‐facing arrows indicating increase and the red down‐facing arrows indicating decrease. Metabolites in grey were not detected in the analysis. (B) Bar graph of the statistically significant and insignificant metabolic changes in the TCA cycle, the urea cycle, and GSH metabolism illustrated as mean normalized fold changes with relative standard deviation. The analytes are sorted by their associated pathways and structural classes highlighted by the color code: 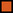 TCA cycle, 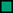 amino acids, 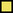 urea cycle, and 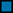 GSH metabolism. The stars above the arrows and bars show the corresponding significance levels ***p ≤ 0.001, **p ≤ 0.01, *p ≤ 0.05 according to Student's *t*‐test (*n* = 3 × 3, 3 biological with 3 technical replicates each). AMP, adenosine monophosphate; ATP, adenosine triphosphate; CoA, coenzyme A; CoA‐NEM, coenzyme A N‐ethylmaleimide derivative; GSH, reduced glutathione; GSSG, glutathione disulfide; GS‐NEM, glutathione N‐ethylmaleimide derivative; NADP^+^/NADPH, nicotinamide adenine dinucleotide phosphate; PP_i_, pyrophosphate.

The TCA cycle and the urea cycle are affected by ( *r* )‐SFN. In the TCA cycle an increase of citrate (1.44‐fold***) and isocitrate (1.38‐fold***) was found, while the other metabolites of the TCA cycle show no changes compared to the solvent control. However, the three amino acids aspartate, asparagine, and proline, which are linked to the TCA cycle, among others, were affected. Aspartate, which can be converted into oxalacetate, is decreased (−0.26‐fold*) (Figure 4A), which might be related to an increased incorporation into the TCA cycle. This assumption is supported by the enrichment of citrate, which is subsequently formed from oxalacetate in the TCA cycle. Oxalacetate, itself could not be used to support a direct relation between changes in aspartate levels and TCA cycle, as it was not detectable. Asparagine, which can form aspartate, is increased (1.33‐fold***) and therefore the equilibrium seems to be on the side of asparagine.

Proline, being decreased (−1.2‐fold***), is linked to the TCA cycle in two ways (Figure 4A). One way is the conversion to pyruvate, which then fuels the TCA cycle via reaction to acetyl‐CoA. This might indicate a flux toward citrate and isocitrate, although the changes of pyruvate and acetyl‐CoA levels are lacking statistical significance (Figure 4B). However, a decrease of CoA, which was detected as its NEM derivative CoA‐NEM (−0.31‐fold*), was observed, which might be the cause or an indication for decreased amounts of acetyl‐CoA.

The other pathway, by which proline is connected to the TCA cycle, is through conversion to glutamate, which can subsequently be converted to oxoglutarate. The strong increase of isocitrate (1.38‐fold***) indicates an inhibition of the isocitrate dehydrogenase, which catalyzes the reaction from isocitrate to oxoglutarate. Such an inhibition may lead to an increased consumption of proline to maintain oxoglutarate levels. This assumption is supported by relatively unchanged levels of the other TCA cycle metabolites including succinate, fumarate, and malate (Figure 4B). However, only a statistical insignificant change of glutamate levels was observed and oxoglutarate, which links glutamate to the TCA cycle, was not detected. (Figure 4B).

### 5.5 Effects of SFN on the Urea Cycle

The strongly decreased proline levels (−1.2‐fold***) might also be related to decreased levels of ornithine (−0.43‐fold***), which is a metabolite of the urea cycle and can be formed from proline (Figure 4A). Furthermore, the already mentioned decrease of aspartate (−0.26‐fold*) might affect the urea cycle, as aspartate reacts with citrulline to form argininosuccinate. To maintain the urea cycle, aspartate consumption may be increased along with a change of argininosuccinate levels, which lack statistical significance (Figure 4B). Additionally, a decrease of *N*‐acetylglutamate (−0.47‐fold**) was observed, which activates the carbamoyl‐phosphate synthetase 1 (CPS I). CPS I is a key enzyme catalyzing the formation of carbamoyl‐phosphate, which then enters the urea cycle. Therefore, the decrease of *N*‐acetylglutamate indicates an inhibition of the urea cycle after ( *r* )‐SFN incubation. *N*‐acetylglutamate has glutamate as a precursor, which in turn can be formed from proline, indicating a connection between their cellular levels. Concerning the described decreases of aspartate and proline, it has to be kept in mind, that these can also be consumed by elevated protein biosynthesis.

### 5.6 Effects of SFN on Purine Metabolism

A further result is the increase of folate (0.65‐fold*), which is linked to the synthesis of purine bases. Since adenine is decreased (−0.38‐fold*) this might be an indicator, that the purine synthesis is affected (Figure S4). However, guanine does not show a statistically significant change compared to the solvent control. The decrease of adenine may also be an explanation for the decrease of adenosine triphosphate (ATP) by −0.43‐fold*, which has been described previously after SFN incubation [16]. Folate is also known to be linked to the synthesis of the pyrimidine base thymine (Figure S4). Therefore, the increase of folate might be connected to the decrease of thymine by −0.37‐fold**. Additionally, a strong decrease of the pyrimidine base cytosine (−1.69‐fold***) was observed, however this is not related to folate levels. The decrease of cytidine is rather connected to the increase of cytidine diphosphate (CDP) by 0.41‐fold**. These decreases of the nucleobases could result in a reduced DNA synthesis followed by a cell cycle arrest, which is described for ( *r* )‐SFN in HepG2 in literature [10].

### 5.7 Effects of SFN on Glycolysis and PPP

Concerning glycolysis and PPP a decrease of total hexose phosphates (hexose‐P) (−0.52‐fold**) and an increase of 6‐phosphogluconate (6‐PG) (0.75‐fold*) were observed, indicating a flux toward PPP. The conversion from glucose 6‐phosphate to 6‐PG is catalyzed by the glucose 6‐phosphate dehydrogenase (G6PD) and yields NADPH (Figure S5). The results match previous findings, where an upregulation of G6PD and a flux from glucose toward ribulose 5‐phosphate was described [16]. NADPH serves as a reducing agent in the cells, thus taking part in the antioxidant response of the cells. It is also used to reduce GSSG to GSH and therefore plays a vital role in glutathione homeostasis. However, NADPH itself was not detected and the changes of NADP^+^ levels did not meet threshold of statistical significance. Therefore, the assumption of elevated NADPH levels was only based on GSH levels and the effects on PPP. The shift from glycolysis toward PPP might also be related to the already mentioned decrease of ATP (−0.43‐fold*). The 1.38‐fold** increase of glycerol 3‐phosphate after SFN incubation may also indicate alterations of the energy metabolism. As a product of lipid degradation, which can fuel glycolysis or gluconeogenesis, the enrichment of glycerol 3‐phosphate might indicate elevated lipid catabolism to support cellular energy demands. The impact on the energy metabolism might also be indicated by the 0.30‐fold* enrichment of uridine diphosphate glucose (UDP‐Glucose). In the first step of glycogen synthesis, it is formed from glucose 1‐phosphate and uridine triphosphate. Therefore, an enrichment of UDP‐glucose might be related to an inhibition of glycogen synthesis, due to the energy deficiency. Another notable alteration, potentially reflecting an impact on energy metabolism, is the −0.38‐fold** decrease of the energy source creatine.

### 5.8 Effects of SFN on GSH Metabolism

To enable reliable quantification of GSH, derivatization with NEM was employed, resulting in the formation of the stable GS‐NEM derivative. Both GS‐NEM and GSSG show increased levels after SFN incubation compared to the solvent control (Figure 4). While the increase of GS‐NEM is 0.97‐fold***, the GSSG is increased 3.96‐fold***. These increases should not be compared directly, because they refer to the solvent control and not to the absolute levels of GSH and GSSG in each sample. The elevated levels of GS‐NEM can be explained by a stimulation of the Nrf2/Keap1 pathway by SFN. Through activation of this signaling pathway, many enzymes are synthesized including GCL, which catalyzes the first step of GSH synthesis [12, 13, 16]. The elevated GSSG levels might subsequently be caused by dimerization of GSH, due to the overall more abundant GSH in the cells.

The decreases of CoA‐NEM (−0.31‐fold*) and cystine (−0.46‐fold*) might be due to SFN redirecting cysteine to GSH synthesis (Figure 4A), as described in the literature [16]. Another explanation for the decrease of cystine is, that it is used by thioredoxin reductase 1 to reduce thioredoxin which participates in various redox reactions, as described in a previous study [16]. As already mentioned SFN is able to directly react with thiols, which might also be related to the decreases of CoA‐NEM and cystine [22].

## 6 Concluding Remarks

The purpose of the current study was to analyze the effects of SFN on the primary metabolism of HepG2 cells using metabolic profiling based on targeted HILIC‐MS/MS analysis. Previous studies have highlighted the pivotal role of reduced glutathione in the context of SFN incubation. However, these investigations did not employ any stabilization methods to preserve this reactive compound during sample processing. Therefore, a thiol derivatization method using NEM was implemented in metabolic profiling for the reliable analysis of reduced glutathione as GS‐NEM and CoA as CoA‐NEM. The frequently mentioned antioxidant properties of SFN were supported by the enrichment of glutathione as well as the elevated flux toward PPP, which might indicate a potentially enhanced NADPH production. These findings support the discussed stimulation of the Nrf2/Keap1 signaling pathway by SFN [12]. While cytoprotective effects of SFN during oxidative stress were not directly tested, these findings indicate that SFN may contribute to such effects.

Moreover, the effects of (R)‐SFN on the levels of several metabolites of the TCA cycle, the urea cycle, the glycolysis, and the PPP were elucidated. The effect of SFN on the TCA cycle is reflected in the enrichment of citrate and isocitrate, which might also be related to the observed changes in the levels of the amino acids aspartate, asparagine, and proline, which can be incorporated into the TCA cycle in different ways. Metabolic profiling data indicate an inhibition in the urea cycle after SFN incubation, due to changes in ornithine and *N*‐acetylglutamate levels, which might also be related to the decrease of proline levels. Furthermore, changes in the levels of folate, adenine, cytidine, and thymine were detected and could lead to reduced DNA synthesis resulting in a cell cycle arrest, which has already been described as an effect of SFN on cancer cells [10]. Concerning energy metabolism, lowered levels of ATP and creatine as well as elevated levels of glycerol 3‐phosphate and UDP‐glucose, were observed, which might indicate an elevated cellular energy demand after SFN incubation.

Overall, this study provides detailed insights into the effects of SFN on the cellular metabolism. The use of a targeted MS/MS approach comes across with limitations in metabolite detections related to the number of simultaneously detectable analytes, occurring matrix effects, and fewer fragmentations of selected molecules especially those with low molecular masses. However, the analyzed metabolites represent the main metabolic pathways, which enables a comprehensive overview over SFN effects on the primary metabolism. Nevertheless, some metabolites, such as oxalacetate, oxoglutarate, and NADPH, amongst others, were not detected in the sample set (Table S4). Therefore, assumptions made concerning these metabolites, were based on the adjacent metabolites in the metabolic pathways. Since the study was carried out using cancer cells in vitro, the differences to primary cells or the whole organism in vivo situation must be considered. Cancer cell lines, used in this study, are regularly characterized by reduced metabolic activity. Also, cancer cells show differences in the glycolysis and PPP compared to primary cells through the Warburg effect [27]. Unlike primary cells, cancer cells possess an impaired oxidative phosphorylation. Consequently, glucose uptake is increased leading to its metabolism predominantly via anaerobic glycolysis, which yields lactic acid. Moreover, the elevated glucose uptake also activates the PPP [27]. Although, the extrapolation of the results concerning these pathways to primary cells and/or the in vivo situation is limited, cancer cells remain suitable for metabolic profiling, because their reduced biological variability enables more consistent and interpretable analysis of complex metabolic data. Nevertheless, additional factors contribute to the assessment of the health‐related properties of SFN for humans. The contents of SFN in broccoli, the absorption through the diet as well as metabolism must be considered, when discussing the effects of SFN in context of human health.

## Conflicts of Interest

The authors declare no conflicts of interest.

## Supporting information




**Supporting File 1**: mnfr70373‐sup‐0001‐SuppMat.docx.

**Supporting File 2**: mnfr70373‐sup‐0002‐SuppMat.xlsx.

## Data Availability

The data that supports the findings of this study are available in the supplementary material of this article.
