# Supplementary material for: Elucidation of Sulforaphane‐Mediated Effects on the Cellular Human Metabolome Using Metabolic Profiling
Source: Mol Nutr Food Res. 2026 Jan 16;70(1):e70373. doi: 10.1002/mnfr.70373 (PMC12810221; doi:10.1002/mnfr.70373)
Supplement: Supplementary file 1 — Supporting File 1: mnfr70373‐sup‐0001‐SuppMat.docx. [file MNFR-70-e70373-s002.docx]

**Supplementary information**

**for**

**Elucidation of sulforaphane-mediated effects on the cellular human metabolome using metabolic profiling**

Nadine Bieß, Hans-Ulrich Humpf, Matthias Behrens, Andrea Gerdemann*

Institute of Food Chemistry, University of Münster, Corrensstraße 45, 48149 Münster, Germany

**Corresponding Author:**

*Dr. Andrea Gerdemann, Institute of Food Chemistry, University of Münster, Corrensstraße 45, 48149 Münster, Germany; email: andrea.gerdemann@uni-muenster.de

**Table S1:** HPLC-quadrupol TOF-MS method for the analysis of the chemical stability of (*R*/*S*)-SFN.

| Mobile phase | A: ACN + 0.1 % FA | | | |
| --- | --- | --- | --- | --- |
|  | B: H_2_O + 0.1 % FA | | | |
| Gradient | Time (min) | Flow (mL/min) | | A % |
|  | 0.00 | 0.400 | | 10 |
|  | 0.50 | 0.400 | | 10 |
|  | 7.00 | 0.400 | | 100 |
|  | 9.00 | 0.400 | | 100 |
|  | 9.10 | 0.400 | | 10 |
|  | 11.00 | 0.400 | | 10 |
| Injection | 10 µL | | | |
| Colum oven | 40°C | | | |
| Source parameters | Source | | HESI | |
|  | Polarity | | positive | |
|  | End Plate Offset | | 500 V | |
|  | Capillary | | 4500 V | |
|  | Nebulizer | | 4.0 bar | |
|  | Dry Gas | | 12 L/min | |
|  | Dry Temp | | 220°C | |
| MS parameter | Mass range | | *m*/*z* 50–1000 | |
|  | Spectra Rate | | 4 Hz | |
|  | Funnel 1 RF | | 150.0 Vpp | |
|  | Funnel 2 RF | | 400.0 Vpp | |
|  | Hexapole | | 150.0 Vpp | |
|  | Ion Energy | | 4.0 eV | |
|  | Low Mass | | *m*/*z* 150 | |
|  | Collision Energy | | 7.0 eV | |
|  | Collision RF | | 650 Vpp | |
|  | Transfer Time | | 80 µs | |
|  | Pre Pulse Storage | | 50 µs | |
| Instrumentation | HPLC  MS | | Elute HT Pump HPG 1300 (Bruker Daltonics)  PAL HTC‑xt auto sampler (CTC analytics)  Elute Column Oven (Bruker Daltonics)  Impact II (Bruker Daltonics) | |

**Table S2:** HPLC-MS/MS method for metabolic profiling.

| Mobile phase | A: ACN/H_2_O (9+1, *v*/*v*) +10 mM NH_4_Ac pH 9 | | | |
| --- | --- | --- | --- | --- |
|  | B: ACN/H_2_O (1+1, *v*/*v*) +10 mM NH_4_Ac pH 9 | | | |
| Gradient | Time (min) | Flow (mL/min) | | A % |
|  | 0.00 | 0.500 | | 100 |
|  | 2.00 | 0.500 | | 100 |
|  | 7.00 | 0.500 | | 25 |
|  | 8.00 | 0.500 | | 25 |
|  | 8.10 | 0.500 | | 100 |
|  | 9.50 | 0.700 | | 100 |
|  | 14.90 | 0.700 | | 100 |
|  | 15.00 | 0.500 | | 100 |
| Injection | 5 µL | | | |
| Colum oven | 40°C | | | |
| Source parameters | Source | | HESI | |
|  | Polarity | | positive/negative | |
|  | Spray Voltage + | | 5000 V | |
|  | Spray Voltage − | | 4500 V | |
|  | Cone Temperature | | 250 °C | |
|  | Cone Gas Flow | | 20 psi | |
|  | Probe Temperature | | 500°C | |
|  | Probe Gas Flow | | 50 psi | |
|  | Nebulizer Gas Flow | | 60 psi | |
|  | Exhaust | | on | |
| Instrumentation | HPLC  MS | | Elute HT Pump HPG 1300 (Bruker Daltonics)  PAL HTC‑xt auto sampler (CTC analytics)  Elute Column Oven (Bruker Daltonics)  EVOQ Elite (Bruker Daltonics) | |

**Table S3:** Analytes included in multi-standard solution at a concentration of 10 µg/mL used for HPLC-MS/MS metabolic profiling.

| **Compound** |
| --- |
| Glucose 1-phosphate |
| Fructose 1,6-bisphosphate |
| 3-Phosphoglycerate |
| Dihydroxyacetone phosphate |
| Glucosamine |
| NADH |
| UDPGA |
| Creatine |
| Fumarate |
| Succinate |
| Aspartate |
| Acetyl-CoA |
| GSH |
| Nicotinate |
| *N*-Acetylglutamate |
| Serotonine |
| Citrulline |
| Glucose |
| Uric acid |
| Adipic acid |
| Biotin |
| NADPH |
| Choline |
| Glucuronic acid |
| Cysteine |
| Pyruvate |
| Fructose |
| *myo*-inositol |
| Ornithine |
| Hypoxanthine |
| Pyridoxal phosphate |
| Nicotinamide |
| Creatinine |
| Urea |
| Dihydrobiopterin |
| Dihydrofolate |
| Levomefolate |
| Biopterin |
| Coenzyme A |

Determination of the cytotoxicity

For the resazurin reduction assay according to a previous publication ^[1]^ 10 000 HepG2 cells/well were seeded in a 96 well plate. The medium was exchanged with serum-free medium after 24 h and after additional 24 h the cells were incubated with (*R*)-SFN. The incubated concentrations were 100 µM, 50 µM, 20 µM, 10 µM, 5 µM, 1 µM and 100 nM and had a final DMSO concentration of 1%. As negative control the cells were treated with 1% DMSO and as positive control T2 toxin (50 µM) was incubated. 10 µL of resazurin (440 µM) were added after 24 h and incubated 120 min at 37°C. The measurement with the microplate reader (Tecan, Gröding, Austria) was performed at 544 nm excitation and 590 nm emission. After correction by a blank the cytotoxicity was calculated relative to the control. The results of the resazurin assay are depicted in Figure S1.

**Figure S1:** Determination of the cytotoxicity of (R)-sulforaphane on HepG2 cells using the resazurin reduction assay. DMSO (1%) was used as negative control and 50 µM T2 toxin was used as positive control. After correction by a blank, the mean viability was calculated relative to the negative control. Significance was calculated according to Student´s T test (***p≤0.001, ** p≤0.01, *p≤0.05; n=3×6, 3 biological with 6 technical replicates each).

**Figure S2:** Reaction of sulforaphane with thiol and amino groups.^[2]^

**Figure S3:** Heatmap of mean metabolic alterations analyzed by targeted HPLC-MS/MS in HepG2 cells after 24 h cysteine deprivation (-Cys) illustrated as fold change to solvent control. Blue boxes indicate enrichment while red boxes indicate depletion of metabolites relative to the control sample. The significance levels are depicted as ***p≤0.001, **p≤0.01, *p≤0.05 according to Student’s t-test (n=3×3, 3 biological with 3 technical replicates each). Abbreviations: glutathione disulfide (GSSG); glutathione N-ethylmaleimide derivative (GS-NEM); pentose phosphate (pentose-P); cytidine diphosphate (CDP); glycerol 3-phosphate (glycerol-3-P).


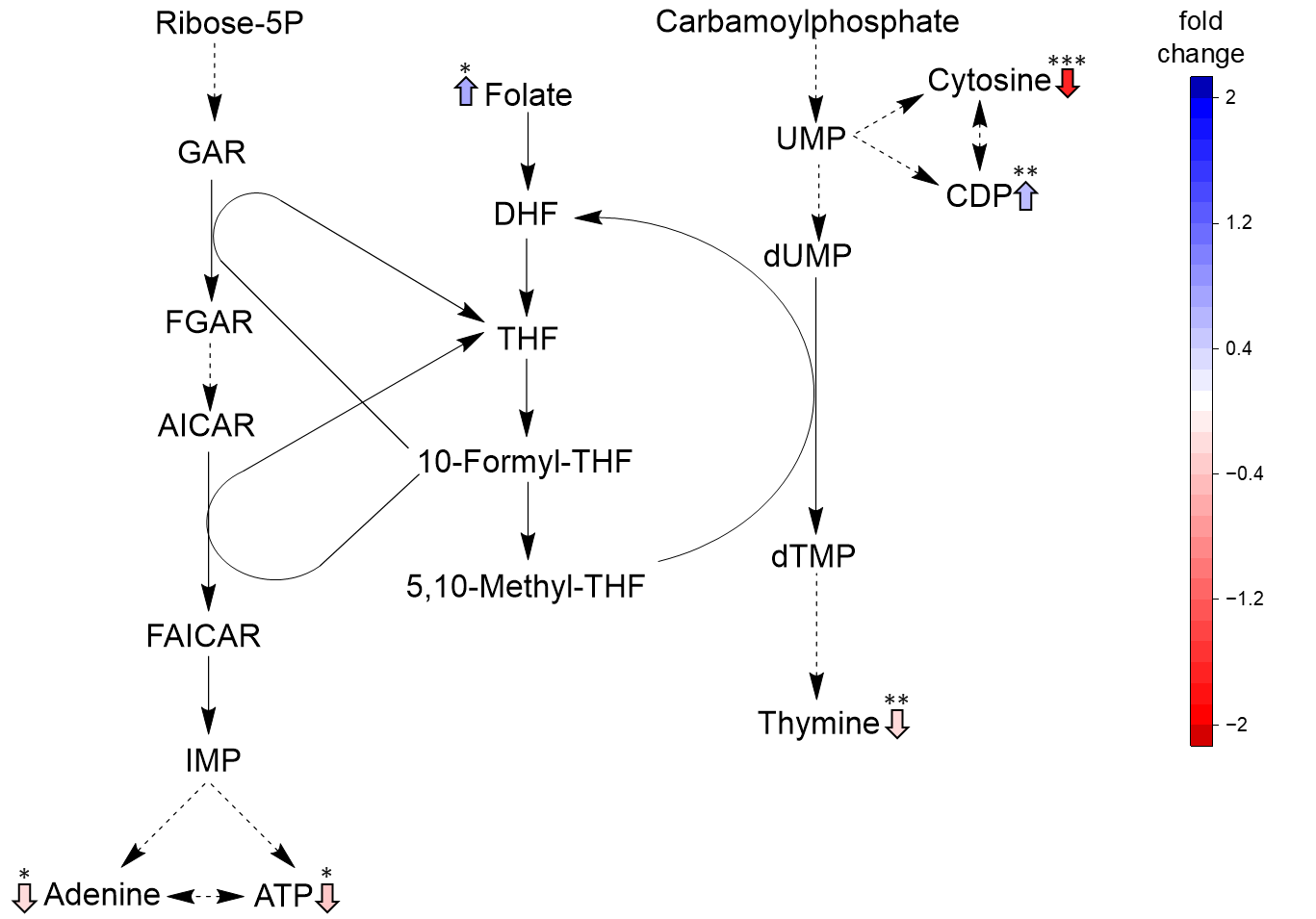


**Figure S4:** Mean metabolic alterations caused by (R)-SFN in purine and pyrimidine synthesis related to folate metabolism analyzed by HPLC-MS/MS. The colored arrows indicate the significant metabolic changes with the blue up-facing arrows indicating increase and the red down-facing arrows indicating decrease. The metabolic changes were calculated as normalized fold changes relative to the control sample. The stars above the arrows and bars show the corresponding significance levels ***p≤0.001, **p≤0.01, *p≤0.05 according to Student’s t-test (n=3×3, 3 biological with 3 technical replicates each). Abbreviations: ribose 5-phosphate (Ribose-5P); glycinamide ribonucleotide (GAR); formylglycinamide ribonucleotide (FGAR); 5-aminoimidazole-4-carboxamide ribonucleotide (AICAR); 5-formylaminoimidazole-4-carboxamide ribonucleotide (FAICAR): inosine monophosphate (IMP); adenosine triphosphate (ATP); dihydrofolate (DHF); tetrahydrofolate (THF); 10-formyltetrahydrofolate (10-formyl-THF); 5,10-methyltetrahydrofolate (5,10-Methyl-THF); uridine monophosphate (UMP); deoxyuridine monophosphate (dUMP); cytidine diphosphate (CDP); deoxythymidine monophosphate (dTMP).


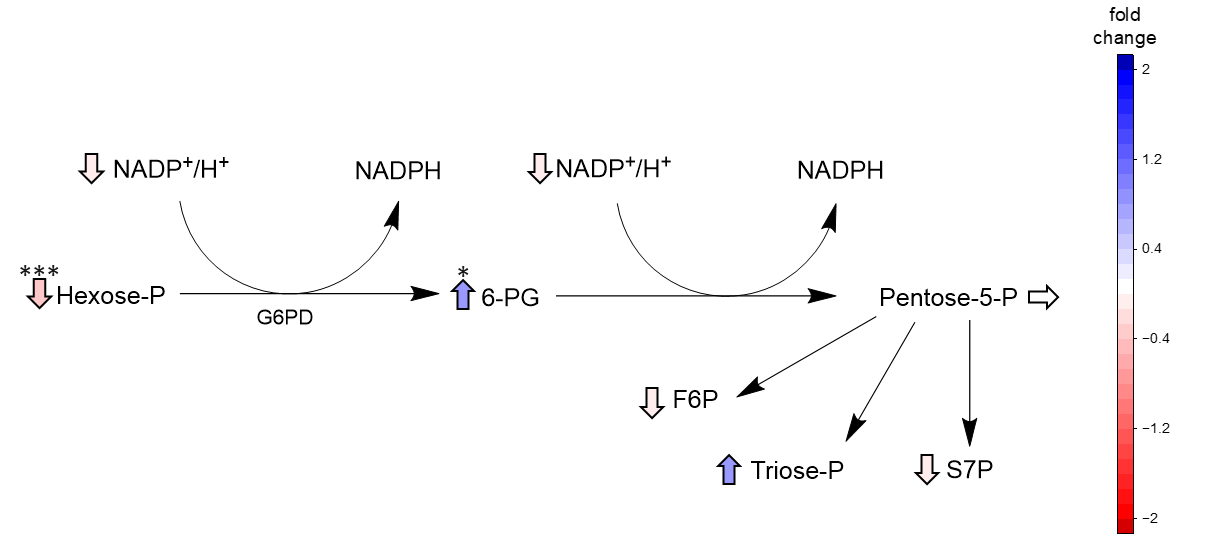


**Figure S5**: Mean metabolic alterations caused by (R)-SFN pentose phosphate pathway analyzed by HPLC-MS/MS. The colored arrows indicate the significant metabolic changes with the blue up-facing arrows indicating increase and the red down-facing arrows indicating decrease. The metabolic changes were calculated as normalized fold changes relative to the control sample. The stars above the arrows and bars show the corresponding significance levels ***p≤0.001, **p≤0.01, *p≤0.05 according to Student’s t-test (n=3×3, 3 biological with 3 technical replicates each). Abbreviations: hexose phosphate (hexose-P); nicotinamide adenine dinucleotide phosphate (NADP^+^/NADPH); glucose 6-phosphate dehydrogenase (G6PD); 6-phosphogluconate (6-PG); pentose 5-phosphate (pentose-5-P); fructose 6-phosphate (F6P); triose phosphate (Triose-P); sedoheptulose 7-phosphate (S7P).

**References**

[1] J. O‘Brien, I. Wilson, T. Orton, F. Pognan, *Eur. J. Biochem.,* DOI: 10.1046/j.1432-1327.2000.01606.x.

[2] F. S. Hanschen, N. Brüggemann, A. Brodehl, I. Mewis, M. Schreiner, S. Rohn, L. W. Kroh, *J. Agric. Food Chem.,* DOI: 10.1021/jf301718g.
